# One-Step Preparation of Fiber-Based Chlorzoxazone Solid Dispersion by Centrifugal Spinning

**DOI:** 10.3390/polym16010123

**Published:** 2023-12-29

**Authors:** Enikő Bitay, Attila Levente Gergely, Zoltán-István Szabó

**Affiliations:** 1 Department of Mechanical Engineering, Faculty of Technical and Human Sciences, Sapientia Hungarian University of Transylvania, Calea Sighișoarei nr. 2, 540485 Târgu-Mureş, Romania; bitay.eniko@eme.ro; 2 Bánki Donát Faculty of Mechanical and Safety Engineering, Óbuda University, Népszínház u. 8, 1081 Budapest, Hungary; 3 Research Institute of the Transylvanian Museum Society, 2–4 Napoca Street, 400009 Cluj-Napoca, Romania; 4 Department of Drugs Industry and Pharmaceutical Management, George Emil Palade University of Medicine, Pharmacy, Science, and Technology of Targu Mures, Gh. Marinescu 38, 540485 Târgu-Mureş, Romania; zoltan.szabo@umfst.ro; 5 Sz-imfidum Ltd., Lunga nr. 504, 525401 Covasna, Romania

**Keywords:** chlorzoxazone, fiber, solid dispersion, solubility enhancement, centrifugal spinning

## Abstract

An amorphous fiber-based solid dispersion of chlorzoxazone was prepared for the first time by employing centrifugal spinning, using polyvinylpyrrolidone as the fiber-forming polymer. After optimization of the spinning parameters, the obtained fibers were characterized using a set of analytical techniques, both in a solid- and solution-state. Morphological characterization revealed a slightly aligned, defect-free fibrous structure with an average fiber diameter of d = 3.07 ± 1.32 μm. The differential scanning calorimetric results indicated a crystalline-to-amorphous transition of the active substance during the centrifugal spinning process, while gas chromatographic determinations revealed a residual ethanol content of 0.42 ± 0.04%. UV spectroscopy indicated the incorporation of chlorzoxazone in the fibrous structures, with an average active substance content of 15.91 ± 0.36 *w*/*w*%. During small-volume dissolution studies, the prepared fiber mats presented immediate disintegration upon contact with the dissolution media, followed by rapid dissolution of the active substance, with 84.8% dissolved at 1 min and 93.7% at 3 min, outperforming the micronized, pure chlorzoxazone. The obtained results indicate that centrifugal spinning is a low-cost, high-yield, viable alternative to the currently used methods to prepare fiber-based amorphous solid dispersions of poorly soluble drugs. The prepared chlorzoxazone-loaded microfibers could be used as a buccal dosage form for the systematic delivery of chlorzoxazone and could potentially lead to a rapid onset of action and longer efficacy of the muscle relaxant drug.

## 1. Introduction

**Figure 1:**
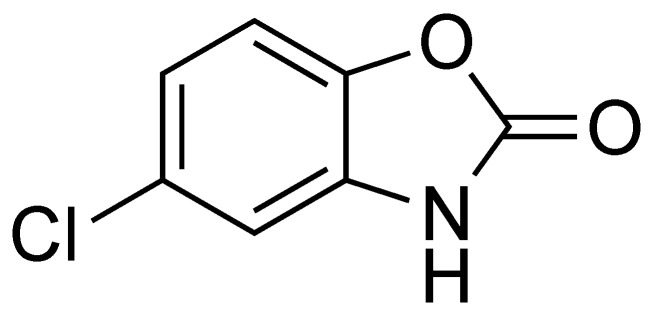
Chemical structure of CLZ.

Chlorzoxazone (5-chloro-3H-1,3-benzoxazol-2-one, CLZ, Figure 1) is a centrally acting muscle relaxant and sedative used in the relief of discomfort associated with acute, painful musculoskeletal conditions [1].

It acts by inhibiting polysynaptic reflex arcs in the spine, which are responsible for inducing and also maintaining muscle spasms [2,3]. The onset of action is within 1 h, it presents a plasmatic half-life of 1–2 h, and the duration of action is up to 6 h [2,4,5]. The principal site of metabolism for CLZ is the liver, where it is rapidly metabolized, mainly by CYP2E1 to 6-hydroxy-CLZ, which is afterward glucuroconjugated and excreted in the urine [4]. Because of its metabolic pathway, CLZ is also an established drug used to assess CYP2E1 activity (phenotyping) by determining the plasma ratio of the parent compound and the 6-hydroxy-CLZ metabolite [6,7,8,9]. The drug is administered orally and generally has minimal side effects (drowsiness, dizziness, and headache being the most common) and no gastrointestinal irritation. However, CLZ has been linked to sporadic cases of serious (including fatal) hepatotoxicity [10,11].

CLZ is characterized by good permeability but low solubility, which inhibits the development of formulations for the buccal administration of CLZ. However, the transmucosal route (including intranasal, buccal, sublingual, and rectal) offers several advantages for drug delivery. Because the oral mucosa is highly vascularized, drugs can be rapidly absorbed and enter directly into the systemic circulation, bypassing the gastrointestinal tract and avoiding first-pass metabolism in the liver. Ease of administration and patient adherence are also among the advantages [12,13]. In the case of CLZ, this would mean that a formulation that offered rapid drug release and increased solubility for the active substance could provide not only rapid onset of the crucial muscle relaxant action for the patients in pain but would also avoid first-pass metabolism in the liver and extend the half-life of the active substance.

Several approaches were employed for the solubility enhancement of CLZ. Moqbel et al. described the preparation of orodispersible tablets using both co-processed excipients such as Pharmaburst^®^500, Starlac^®^, Pearlitol flash^®^, Prosolv^®^ ODT, and F-melt^®^, but also by liquisolid techniques, employing Avicel PH101, Microcelac 100, and Cellactose 80 as carriers [14,15]. The preparation of solid dispersions with Pluronic F127 was also described [16], as well as the co-grinding of the active substance with different carriers [17]. In this case, the best results were obtained with PEG 4000, which could be afterward compressed into a tablet dosage form. Inclusion complexes were also prepared with β- and hydroxypropyl-β-cyclodextrins using freeze-drying. The authors concluded that the latter cyclodextrin derivative conferred better solubility and dissolution rate when compared to the pure active ingredient, their physical mixture, and also the inclusion complex prepared with β-cyclodextrin [18].

The solubility of poorly soluble active substances can also be increased by the development of amorphous solid dispersions (ASDs). ASDs are molecular mixtures of drugs with low water solubility and hydrophilic carriers. The preparation of ASDs requires the dispersion of the active substance within a polymeric matrix, which inhibits aggregation and stabilizes it in the amorphous state [19,20,21]. The prepared amorphous form is in a higher energy state when compared to the crystalline form; thus, it dissolves more rapidly. Several approaches were developed for the preparation of ASDs, but fundamentally, they rely on the breakage of the lattice structure of the crystalline-active substance by converting it into a liquid state (generally by melting or dissolving it in a suitable solvent) and generating an amorphous state by rapid cooling or drying [20]. Thus, ASD preparation methods can be classified as melting or fusion-based and solvent-based methods. One of the rapidly growing, popular solvent-based techniques to prepare ASDs is electrospinning. In this technique, high electrical energy is employed for the rapid evaporation of the solvent, which is more efficient when compared to simple evaporation [22]. In detail, this technique generally involves the use of a volatile solvent and the preparation of a polymeric solution in which the active substance is dissolved. The obtained solution is ejected from a metal spinneret under a high electric field and flies toward the grounded collector. During this flight, the fluid jet solidifies, and non-woven mats form, consisting of nanoscale fibers with high porosity and a large surface-to-volume ratio.

Although the production of nanofibers with electrospinning is widespread and presents several advantages, widespread commercial use of the method is hindered by low production rates and costs. Centrifugal spinning is a simpler, low-cost, and high-production yield alternative to electrospinning [23,24]. In this case, the electrostatic force needed for fiber production is replaced by centrifugal forces. It enables high production rates of fibrous material with low costs. Moreover, the technique is not sensitive to the conductivity and electric properties of the spinning solution, broadening the spectrum of solutions that can be spun into fibers. During fiber production, the spinning fluid is fed into a rotating spinneret, which contains multiple nozzles. Above a critical rotation speed, the spinning solution is forced to move outward, passing through the nozzle; thus, a jet forms. Due to the centrifugal force and air friction, the jets become elongated, and this leads to the formation of fibers, which are deposited on the collectors [23,24,25,26]. 

In the present study, for the first time, centrifugal spinning was used in the preparation of CLZ-loaded fibrous ASD. Optimization of the centrifugal spinning process was undertaken, and the obtained fibrous material was examined in a solid state through morphological characterization and thermal analysis. The active substance and the residual solvent content of the prepared fibers were also determined, as well as the dissolution characteristics. 

## 2. Materials and Methods

### 2.1. Materials

Poly(vinyl-pyrrolidone) (PVP K90F, Kollidon K90F) was obtained from Sigma Aldrich (Merck, Darmstadt, Germany), while ethanol (EtOH, 96%) was from the Chemical Company (Iasi, Romania). Chlorzoxazone (CLZ) was provided as a gift sample by a local pharmaceutical company from Targu-Mures, Romania. Hydrochloric acid 36% (*w*/*w*), phosphoric acid (98%), and potassium-dihydrogen phosphate was ordered from Merck (Darmstadt, Germany). Ultrapure water was obtained by the Barnstead Nanopure Diamond water purification system (Boston, MA, USA). All materials were used as received without further purification. 

### 2.2. Solution Preparation

To optimize the centrifugal spinning process, PVP solutions with different concentrations were prepared. Thus, the required amounts of PVP to prepare 20, 25, and 30 *w*/*w*% solutions were dissolved in EtOH through stirring with a magnetic stirrer (ARE 1500, Fisher Scientific, Waltham, MA, USA) at 750 rpm for 1 h. The process resulted in a clear, transparent, viscous polymer solution.

After the optimization study, CLZ containing the PVP polymeric solution was prepared by first dissolving 0.234 g of CLZ in 3.75 g of EtOH with a magnetic stirrer for 10 min at 750 rpm and then adding 1.25 g of PVP. Thus, the PVP to EtOH concentration was set to 25 *w*/*w*%, and the CLZ concentration was set to 15.77 *w*/*w* in the dry PVP fiber-based solid dispersion. After 1 h of stirring, a clear solution was obtained. 

### 2.3. Fiber Production

**Figure 2:**
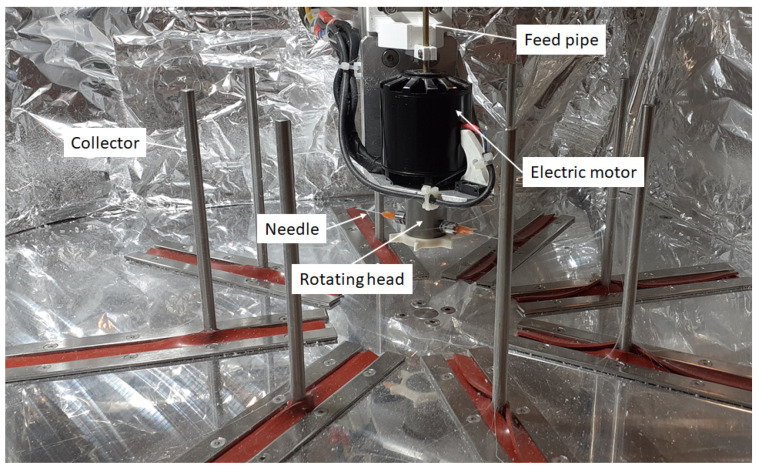
Custom-built centrifugal spinning machine.

Fiber production was carried out on a custom-built centrifugal spinning machine. Figure 2 shows the centrifugal spinning setup containing 8 collector rods set on the circumference of a circle. The diameter of the circle can be set so that the desired distance between the needle and the collector can be achieved. The rotating head holds 2 needles via two thread-to-luer connectors. The rotating head is rotated at a specific speed by the electric motor. The rotational speed of the electric motor can be varied in the range of 2000–10,000 rpm. The solution is fed into the rotating head through the aid of PTFE tubing located in the copper pipe that passes through the hole found in the shaft of the electric motor. This setup provides the possibility to prepare fiber safely and continuously.

The centrifugal spinning experiments were conducted using G23 needles, a 100 mm collector-to-needle distance, and a 60 mL/h flow rate that was provided by a syringe pump (KD Scientific, Holliston, MA, USA). The experiments were performed under standard laboratory conditions at room temperature (~20 °C) and at ~60% relative humidity.

### 2.4. Morphological Characterization of the Fibers by Scanning Electron Microscopy (SEM)

The morphology and average fiber diameter of the prepared fiber mats were examined with a scanning electron microscope, JEOL JSM-6380LA (JEOL, Tokyo, Japan). Due to the centrifugal production process, the fiber mats are rather fluffy; thus, Au coating was necessary to obtain adequate SEM images. Sputter coating was carried out on a JEOL JFC-1200 sputter-coater using Ar gas. The open-access ImageJ software (version 1.52a, National Institutes of Health, Bethesda, MD, USA) was used to measure 100 fibers at different parts of the samples to determine the average fiber diameters and standard deviations with the equations of the normal distribution function.

### 2.5. Determination of CLZ Solubility

The solubility of CLZ was determined in three different media, as follows: water, 0.1 M HCl, and 0.1 M phosphate buffer pH 6.8. An excess amount of CLZ (approx. 5 mg) was suspended in 5 mL of media and magnetically stirred for 24 h at 600 rpm using a JK SMS HS magnetic stirrer (JKI, Shanghai, China) and Teflon-coated stir bars. After stirring, the suspensions were filtered using 0.45 μm syringe filters. The CLZ concentration was determined at λ_max_ = 280 nm using a Shimadzu UV-1601PC spectrophotometer (Shimadzu Corporation, Kyoto, Japan) after an appropriate dilution with water. The solubility measurements were performed in triplicate for each media.

### 2.6. Solid-State Characterization of the Fibers Using Differential Scanning Calorimetry (DSC)

Thermal measurements were performed on a Shimadzu DSC-60 (Shimadzu, Tokyo, Japan) differential scanning calorimeter. Samples of 3–10 mg were placed into aluminum pans and the pans were sealed, and an empty aluminum pan of the same type was used as a reference. Samples were subjected to heating in the temperature range of 30 to 220 °C with a heating rate of 5 °C/min under an air atmosphere.

### 2.7. Determination of Residual Ethanol Content of the Fibrous Mats Using Gas Chromatography (GC)

The content of ethanol as a residual solvent in the fibrous samples was measured on a Dani Master GC Gas Chromatograph with a flame ionization detector (FID) (Dani Instruments S.p.A, Milan, Italy). An Optima WAX capillary column was used for the measurements (adsorbent thickness, 2 μm; diameter, 0.53 mm; length, 30 m), employing the following temperature gradient program: 60 °C for 5 min, increase to 90 °C with a rate of 5 °C/min; hold for 5 min. The temperature of the FID was set to 250 °C, whereas the rest of the parameters were as follows: injector temperature, 250 °C; injection volume, 1 μL; carrier gas, N_2_ (10 mL/min); combustion gases: H_2_ (40 mL/min), synthetic air (220 mL/min), and N_2_ (25 mL/min).

### 2.8. Determination of CLZ Content of the Fibrous Samples by UV Spectroscopy

UV spectroscopy was employed for the determination of the active substance content of the obtained fibers. The UV absorbance of the samples was determined at λ_max_ = 280 nm using a Shimadzu UV-1601PC spectrophotometer (Shimadzu Corporation, Kyoto, Japan). First, approx. 20 mg of fibrous sample was dissolved in 50 mL of water and further diluted afterward with the same solvent to achieve a final theoretical concentration of approx. 21 μg/mL. The active substance content of the fibers was determined in triplicate based on a previously constructed calibration curve.

### 2.9. Small-Volume In Vitro Dissolution Studies

Small-volume dissolution studies were performed as described in our earlier publication in an in-house-built custom setup [27]. Briefly, dissolution tests were performed in 2.7 cm ID × 11.5 cm glass tubes immersed in a water bath maintained at 37 ± 1 °C using an Erweka ET 1500I immersion thermostat (Erweka GmbH, Heusenstamm, Germany). An amount of 20 mL of 0.1 M phosphate buffer was used as dissolution media, and magnetic stirring was performed using a JK SMS HS magnetic stirrer (JKI, Shanghai, China) placed under the water bath and with Teflon-coated stir bars located inside the glass tubes. The stir rate was set to 200 rpm, and the dissolution process was followed for 30 min. At predetermined time points (1 min, 3 min, 5 min, 10 min, 15 min, 30 min), 1 mL samples were withdrawn and filtered through a 10 µm Whatman filter and, if necessary, further diluted with the dissolution media before UV spectroscopic analysis by using the same conditions as described earlier (Section 2.8).

## 3. Results and Discussion

### 3.1. CLZ Solubility

The central muscle relaxant, CLZ is considered by the Biopharmaceutics Classification System (BCS) as a class II drug, displaying low solubility and high permeability. The literature data reveal that the water solubility of CLZ is determined at 0.2–0.3 mg/mL [1]. The physical characteristics of the active pharmaceutical ingredients can also alter their solubility. The exact solubility value of the received micronized sample of CLZ was determined in three different media. The results obtained from triplicate measurements are summarized in Table 1.

As the results indicate, the solubility of CLZ was the lowest in acidic media and showed comparable values in water and 0.1 M phosphate buffer with a pH of 6.8. The results are in correlation with the acid-base properties of CLZ, as it is a weak acid with a pKa value of around 8.25 [28], thus displaying lower solubility at acidic pH values.

As CLZ is a BCS class II drug [17], its bioavailability is likely to be dissolution rate-limited [29], and an increase in solubility would hypothetically also increase its bioavailability. Thus, the preparation of CLZ-containing amorphous solid dispersions was undertaken to enhance the solubility of the active substance.

### 3.2. Optimization of Centrifugal Spinning Parameters through Fiber Morphology

Since there is no literature data regarding the preparation of solid dispersions of CLZ prepared through centrifugal spinning, only limited information was available regarding the overall spinnability of different drug-loaded polymeric solutions and the influence of various parameters upon the fiber morphology.

**Figure 3:**
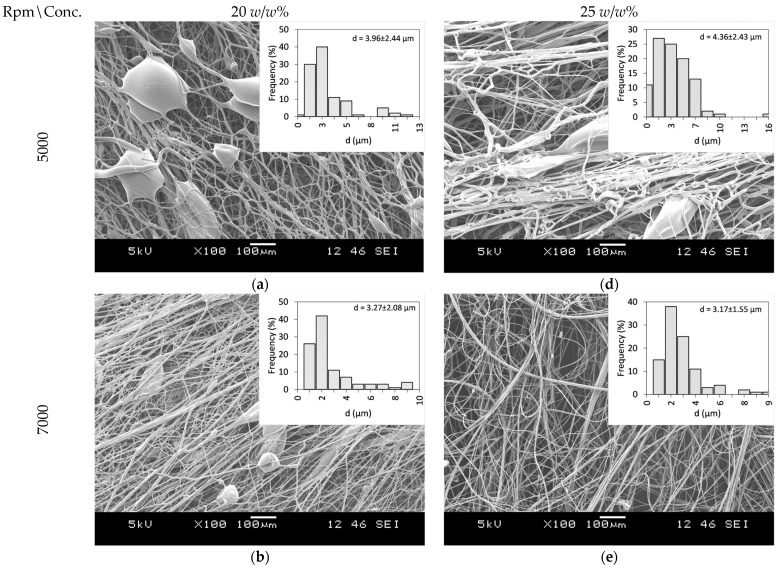

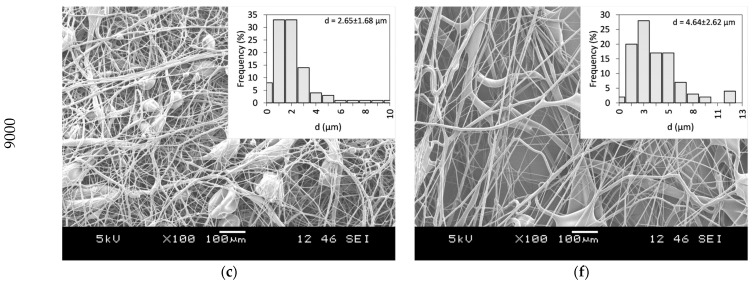
SEM Images of PVP fiber mats acquired at different concentrations and rotational speeds: 20 *w*/*w*% PVP solution at (**a**) 5000 rpm, (**b**) 7000 rpm, and (**c**) 9000 rpm, and 25 *w*/*w*% PVP solution at (**d**) 5000 rpm (**e**) 7000 rpm, and (**f**) 9000 rpm.

PVP as a fiber-forming polymer was successfully used in our previous publications, both in electrospinning and centrifugal spinning [30,31,32,33]. Thus, the same polymer, with a high molecular weight (PVP K90), was selected, and ethanol was chosen as a non-toxic, widely available solvent that dissolves both the PVP and CLZ in satisfactory concentrations. The centrifugal spinning process was optimized, and the spinnability, average fiber diameter, and morphology of the fiber mats were determined for different PVP concentrations. Three different rotation speeds (5000, 7000, and 9000 rpm) were evaluated. SEM images of the prepared PVP fiber mats are presented in Figure 3. A general summary of the obtained results is presented in Table 2.

Fibers were obtained when using 20 and 25 *w*/*w*% ethanolic PVP solutions at all the tested spinning speeds. Using a 30 *w*/*w*% PVP solution concentration did not result in fiber formation, regardless of the rotational speed applied. Bead-free fibers were obtained only using 25 *w*/*w*% PVP at 7000 rpm, while both lower and higher rotational speeds led to bead formation.

Fiber formation requires a balance between the surface tension of the used solution and the stresses acted upon the fiber during fiber formation. The stresses arise as a combination of the tensile force generated by the centrifugal force and the viscoelastic forces due to the inherent nature of polymeric solutions and material. It has been observed that as the solution concentration increases, bead formation reduces due to the stress increase resulting from the increase in the viscoelastic forces [34]. In the case of centrifugal spinning, the rotational speed also has a significant effect on the bead formation since the acting centrifugal forces are a function of the rotational speed. As the rotational speed increases, the combined effect of the forces results in a stress that is higher than the surface tension of the solution; thus, bead formation occurs.

**Figure 4:**
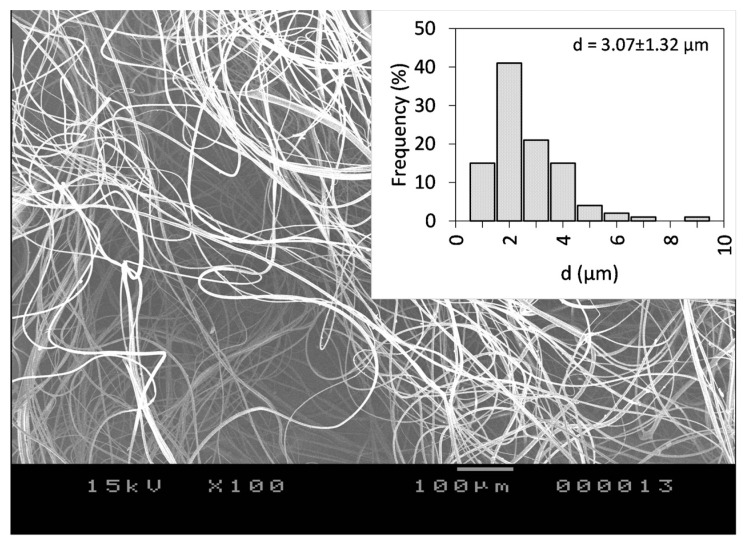
SEM images of the CLZ-loaded PVP fiber mat obtained by centrifugal spinning using 25 *w*/*w*% PVP concentration with respect to the solvent, 7000 rpm rotational speed, G23 needles, 100 mm collector-to-needle distance, and 60 mL/h flow rate at 100× magnification.

As a next step, the incorporation of CLZ into the ethanolic PVP-based spinning solution was employed. The CLZ concertation was set to 15.77 *w*/*w*% with respect to the PVP amount, whereas the PVP concentration was set to be 25 *w*/*w*% with respect to the used EtOH amount. Using the optimal centrifugal spinning conditions, CLZ-loaded PVP-based non-woven fiber mats were prepared. The SEM image of the CLZ-loaded PVP fiber mat can be seen in Figure 4.

The fibers were randomly oriented and bead-free, with an average fiber diameter of d = 3.07 ± 1.32 μm, virtually identical to the diameter obtained for the drug-free fibers, an indication that the CLZ does not affect the surface tension of the solution significantly. The calculated theoretical concentration of CLZ was 15.77 *w*/*w*% of dry fiber. Fiber production lasted for 6.5 min and a total of 694 mg of the fibrous sample could be collected. We normalized the production rate to 1 h, resulting in 6.4 g of dry fibers, which is around two orders of magnitude higher than the production rate of the electrospinning process.

### 3.3. Thermal Characterization of the Obtained Fiber Mats

**Figure 5:**
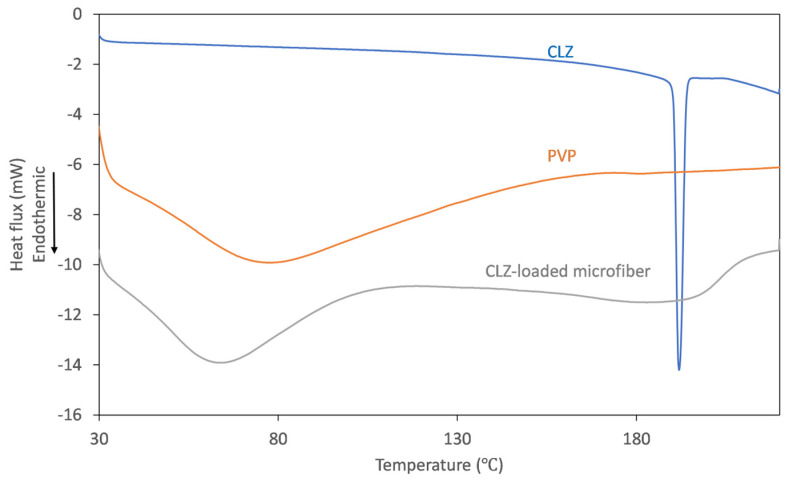
DSC thermograms recorded for CLZ (blue), PVP K90 (orange), and CLZ-loaded PVP microfibers (gray).

To track the physicochemical changes that occur through centrifugal spinning, solid-state characterization of the obtained fibrous mats was undertaken using thermal analysis. The DSC thermograms obtained for the individual components and the obtained microfibers are represented in Figure 5.

On the thermogram recorded for CLZ, the presence of a single, sharp-melting endotherm can be observed at 191.9 °C, indicating its crystalline nature. The obtained value is in good agreement with the data reported previously [14]. The thermogram of the fiber-forming amorphous PVP is characterized only by a broad endothermic event below 110 °C, corresponding to the dehydration process of the hygroscopic polymer. Upon analyzing the thermogram of the CLZ-loaded fiber mats, it can be observed that the broad endothermic peak shifted to slightly lower temperatures, which could be caused by residual ethanol. Interestingly, the melting endotherm of the active ingredient was absent, indicating that a crystalline-to-amorphous transition of CLZ occurred during centrifugal spinning.

### 3.4. Drug Content and Residual Solvent of the Prepared Microfibers

**Figure 6:**
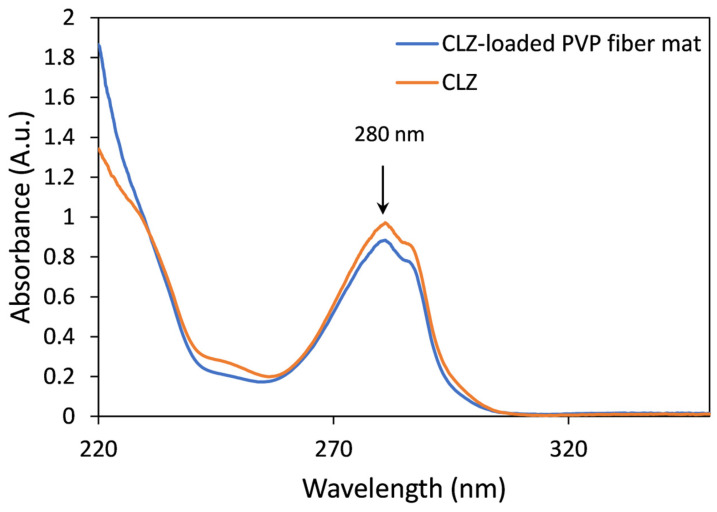
Representative UV spectra of CLZ (orange) and the CLZ-loaded PVP fiber mat (blue).

The CLZ concentration of the prepared fibrous mats was determined by UV spectroscopy. Comparative UV spectra obtained for solutions of CLZ and fibrous samples were similar, and interferences or spectral shifts were not observed. The only differences were found in the low UV region due to the presence of PVP (Figure 6).

Based on the UV spectroscopic measurements, the drug-loaded microfibrous mats contain 15.91 ± 0.36 *w*/*w*% CLZ, which corresponds to 100.89 ± 2.28% of the theoretically calculated value.

Since alcoholic solutions were used for centrifugal spinning, the determination of ethanol as a residual solvent was also performed using GC. The current regulatory guidelines classifies ethanol as a low-toxic potential solvent (Class 3) with a maximum upper limit of 0.5%. The GC determinations revealed that the prepared fibrous mat had a residual ethanol content of 0.42 ± 0.04%. Although the obtained values were under the upper limit stipulated by authorities, when compared with our previous publication, when similar, microfibrous PVP-based ethanolic solutions were used, the currently obtained results are higher (<0.10% using electrospinning vs. 0.42% using centrifugal spinning), meaning that electrospinning led to rapid and more complete solvent evaporation when compared to centrifugal spinning.

### 3.5. In Vitro Dissolution Studies

**Figure 7:**
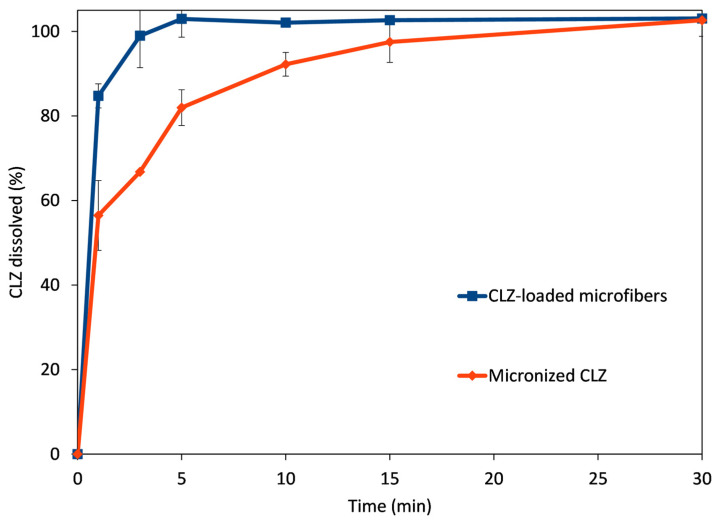
Comparative dissolution profiles obtained for CLZ-loaded microfibers (blue trace) and micronized CLZ (red trace).

To compare the performance of the CLZ-loaded microfibrous mats to the micronized active, small-volume in vitro dissolution studies were performed using 0.1 M phosphate buffer, pH 6.8. The obtained dissolution profiles are represented in Figure 7.

The microfibrous mat disintegrated when it came into contact with the dissolution medium, rapidly releasing the active substance CLZ. The rapid disintegration was facilitated by the hydrophilic polymer, together with the high porosity and high surface-to-volume ratio due to the fibrous structure. The amorphization of CLZ during the centrifugal spinning process also led to rapid solubilization. The combined results of these effects are revealed at the first time points of the comparative dissolution profiles. The rapid disintegration and release of CLZ led to an almost immediate and complete dissolution of the active substance (84.8% at 1 min and 93.7% at 3 min). In comparison, only 62.3% and 67.1% of the active substance was solubilized at 1 and 3 min, respectively, while full solubilization of the micronized active substance was achieved only at 30 min.

## 4. Conclusions

Centrifugal spinning was successfully employed to produce PVP-based ASD of CLZ. Solid-state characterization of the non-woven mats revealed a microscale, randomly oriented, homogenous fibrous structure. During the centrifugal spinning process, due to the rapid evaporation of the solvent, the amorphization of CLZ could be revealed by thermal analysis. GC analyses also revealed that, when compared to electrospinning, the product obtained by centrifugal spinning contained a higher concentration of residual ethanol; however, its concentration was still below the limit set by regulatory authorities. UV spectroscopic measurements confirmed the incorporation of the active substance into the fibrous structure, while the in vitro dissolution studies confirmed an instant disintegration of the fibers and rapid release and solubilization of the active substance. The results indicate that centrifugal spinning is a cost-effective way of producing ASDs and can be a promising technique to obtain a buccal formulation of the muscle relaxant CLZ, which could provide a rapid onset of action and a longer therapeutic effect if absorbed from the oral cavity.

## Figures and Tables

**Table 1:** Solubility of chlorzoxazone in different media.

Media	Solubility (μg/mL) *
0.1 M HCl	322.5 ± 20.8
Water	517.9 ± 4.5
0.1 M phosphate buffer pH 6.8	492.7 ± 14.6

* Average value ± standard deviation (n = 3).

**Table 2:** Effect of PVP concentration and rotational speed on fiber diameter and morphology.

PVP Concentration (*w*/*w*%)	Rotational Speed (rpm)	Fiber Diameter (μm)	Presence of Beads
20%	5000	3.96 ± 2.44	Yes
	7000	3.27 ± 2.08	Yes
	9000	2.65 ± 1.68	Yes
25%	5000	4.36 ± 2.43	Yes
	7000	3.17 ± 1.55	No
	9000	4.64 ± 2.62	Yes
30%	5000	-	-
	7000	-	-
	9000	-	-

## Data Availability

Data are contained within the article.
